# The dynamic evolution of mobile open reading frames in plastomes of *Hymenophyllum* Sm. and new insight on *Hymenophyllum coreanum* Nakai

**DOI:** 10.1038/s41598-020-68000-7

**Published:** 2020-07-06

**Authors:** Hyoung Tae Kim, Jung Sung Kim

**Affiliations:** 1 Institute of Agricultural Science and Technology, Chungbuk National University, Cheongju, Chungbuk 28644 Korea; 2 Department of Forest Science, Chungbuk National University, Cheongju, Chungbuk 28644 Korea

**Keywords:** Evolution, Plant sciences

## Abstract

In this study, four plastomes of *Hymenophyllum*, distributed in the Korean peninsula, were newly sequenced and phylogenomic analysis was conducted to reveal (1) the evolutionary history of plastomes of early-diverging fern species at the species level, (2) the importance of mobile open reading frames in the genus, and (3) plastome sequence divergence providing support for *H. coreanum* to be recognized as an independent species distinct from *H. polyanthos*. In addition, 1C-values of *H. polyanthos* and *H. coreanum* were measured to compare the genome size of both species and to confirm the diversification between them. The *rrn16-trnV* intergenic regions in the genus varied in length caused by Mobile Open Reading Frames in Fern Organelles (MORFFO). We investigated enlarged noncoding regions containing MORFFO throughout the fern plastomes and found that they were strongly associated with tRNA genes or palindromic elements. Sequence identity between plastomes of *H. polyanthos* and *H. coreanum* is quite low at 93.35% in the whole sequence and 98.13% even if the variation in *trnV-rrn16* intergenic spacer was ignored. In addition, different genome sizes were found for these species based on the 1C-value. Consequently, there is no reason to consider them as a conspecies.

## Introduction

Plastomes of vascular plants are varied in size (120–170 kb) and compact with 110–130 genes^1–5^. In angiosperms, they are normally conserved in sequence, gene contents and organization^1^ except in heterotrophic plants^6,7^ or specific lineages^8–10^. Consequently, single nucleotide polymorphisms (SNPs) and insertions/deletions (indels) among infraspecific taxa or individuals in angiosperms are very low^11–14^. In contrast to angiosperms, the genetic variation in plastomes between individuals in ferns is almost similar to the interspecific genetic variation in angiosperms (e.g., for *Equisetum arvense*^15^). This is likely due to the longer evolutionary history of ferns than angiosperms.

Since the first plastome sequence of *Adiantum capillus-veneris* L. was reported^16^, plastomes from 11 orders of extant ferns^17^ have also been sequenced^18–22^. These data have given insights into (1) phylogenomics for resolving the deep relationships throughout fern lineages^19,23,24^, (2) inserted foreign DNA^3,25^, and (3) lineage-specific structural evolutions^3,26,27^. However, because a large proportion of plastome sequence data in ferns belongs to the order Polypodiales, which is the most derived order in the extant ferns^17^, the evolution of plastomes at a low taxonomic level in eusporangiate ferns and basal leptosporangiate ferns is still equivocal.

Hymenophyllaceae, filmy ferns, is a basal family of leptosporangiate ferns^17^, and it consists of more than 600 species^28^. Their distinctive feature, single-cell thick laminae, easily distinguishes them from other fern families^29^. However, infra-familial classification of the filmy ferns has been argued for a long time^30–33^. Nevertheless, Hymenophyllaceae is traditionally classified into two major clades, “trichomanoid” with obconic or tubular involucre and “hymenophylloid” with bivalvate involucre. This relationship has also been clarified using recent molecular analysis^28^.

*Hymenophyllum s.l.* is comprised of more than 300 species with a nearly cosmopolitan distribution^34^. Pryer et al.^28^ showed that *Serpyllopsis*, *Cardiomanes*, and *Microtrichmanes*, which were segregated from each other or belonged to *Trichomanes* by transitional classifications^30,32,33,35^, were included within *Hymenophyllum s.l.* Although, they also mentioned that their data set was insufficient in terms of taxon sampling and the number of genes used to evaluate all of the segregates within *Hymenophyllum s.l.* Hennequin et al.^36^ reconstructed the phylogenetic tree of *Hymenophyllum* s.l. using increased sampling and additional genes and divided *Hymenophyllum s.l.* into eight subgenera. Interestingly, four segregate genera and one section belonging to *Trichomanes* formed a clade with *Hymenophyllum* species, in agreement with Pryer et al.^28^. Recently, Ebihara et al.^29^ divided *Hymenophyllum s.l.* into 10 subgenera based on molecular phylogenetic analyses and macroscopic characters.

**Figure 1:**
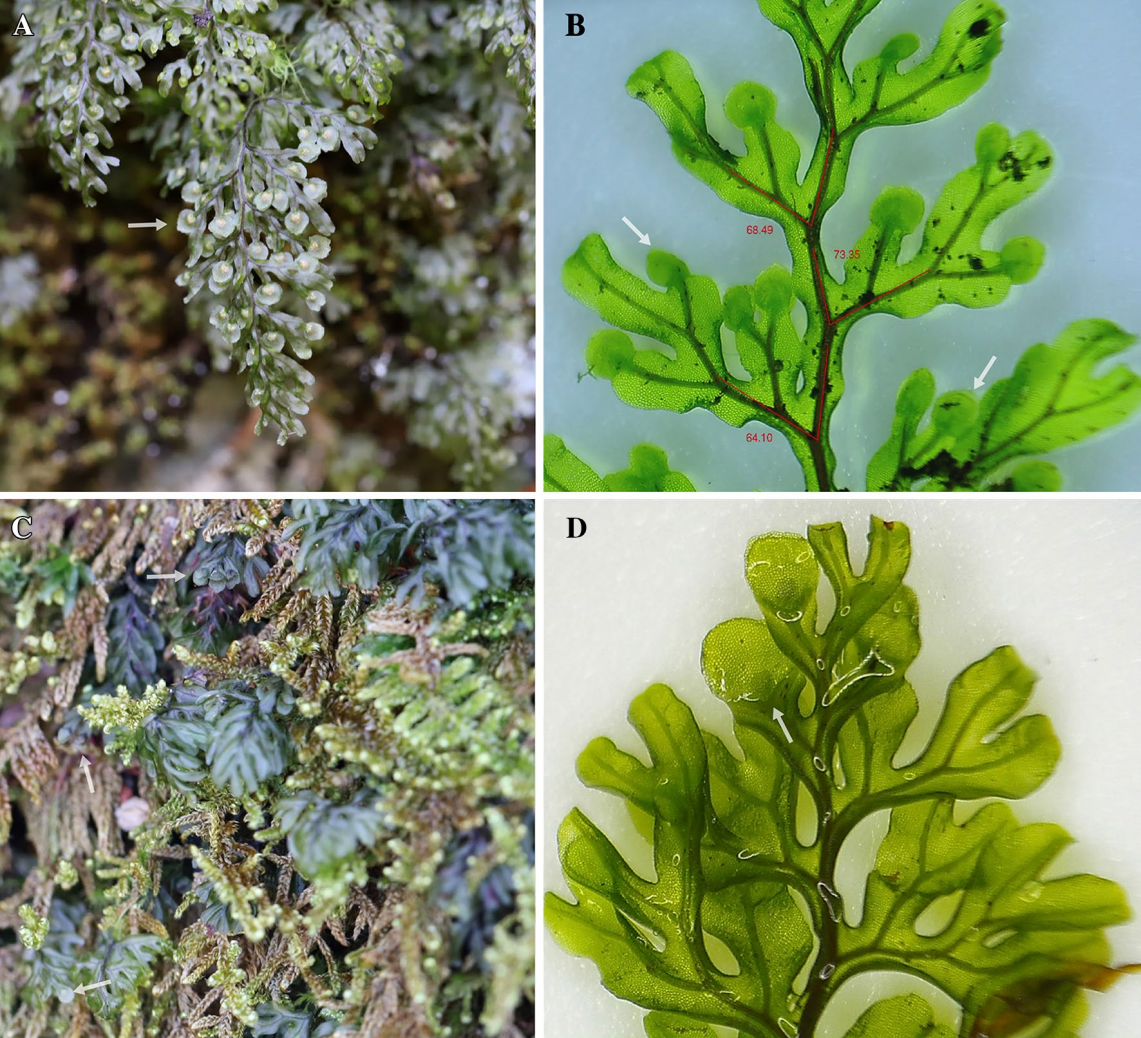
Phenotypes of *Hymenophyllum polyanthos* (**A** and **B**) and *H. coreanum* (**C** and **D**). (**A**) Photo of a whole plant of *H. polyanthos* in the field. (**B**) Microphotograph of *H. polyanthos.* (**C**) *H. coreanum* in the field*.* (**D**) Microphotograph of *H. coreanum*. White arrow refers to the involucre and red angle refers to angle from rachis to pinna.

In Korea, three species and one unacceptable taxon of the genus *Hymenophyllum* have been reported: *Hymenophyllum barbatum* (Bosch) Baker, *H. polyanthos* (Sw.) Sw, *H. wrightii* Bosch and *H. coreanum* Nakai. Among them, *H. coreanum* was first recognized by Nakai^37^ who collected the sample from Kumgangsan in Gangwondo, Korea and described it as a plant with 2–5 mm stipes, 5–15 mm fronds and dense sori in apex fronds. In contrast to *H. coreanum*, *H. polyanthos* in Korea is 1.5–3 times longer and has involucres from the apex to the middle of the frond (Fig. 1)^38^. However, in spite of these different characteristics, *H. coreanum* has been considered a synonym of *H. polyanthos* by many researchers. The broadly described species, *H. polyanthos* has a worldwide distribution but probably includes a number of distant lineages in the subgenus *Mecodium* that do not have any specialized morphological characters^39^. The recent phylogeny of *Hymenophyllum s.l.* using molecular markers revealed that *H. polyanthos* was highly polyphyletic^36,40^. Therefore, more clear scientific evidence for considering the correct taxonomical position of *H. coreanum* and its relationship with *H. polyanthos* is necessary.

In this study, we sequenced four new plastomes of *Hymenophyllum* species distributed in the Korean Peninsula: (1) to explore the plastome evolution of early-diverging leptosporangiate ferns at the species level, (2) to verify the Mobile Open Reading Frames in Fern Organelles (MORFFO) which were identified by Robison et al.^25^, and (3) to investigate the possibility that the two morphologically similar species, *H. polyanthos* and *H. coreanum*, are distinct species. In addition, 1C-values of both species were measured to compare genome size and to explore the diversification between *H. polyanthos* and *H. coreanum*.

## Results

### Genome structure and gene contents of plastomes in *Hymenophyllum*

**Figure 2:**
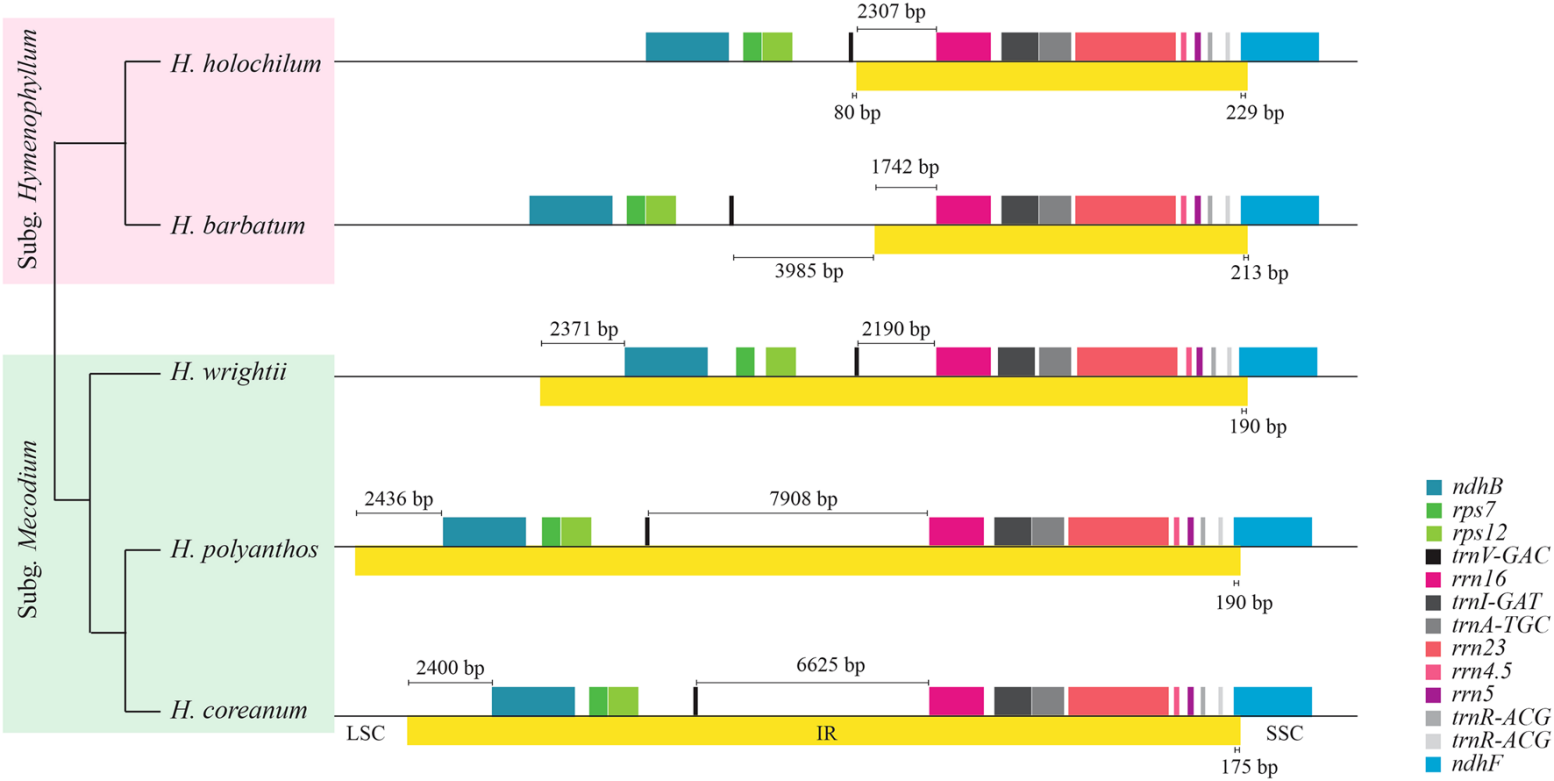
IR boundaries in the plastomes of *Hymenophyllum*. The phylogeny of *Hymenophyllum* species is extracted from the phylogeny of Hymenophyllaceae using 85 genes in this study. The boxes above the black line refer to the genes and the yellow box below the line refers to the region.

The newly sequenced plastomes of the four *Hymenophyllum* species were from 144,112 bp to 160,865 bp in length with a GC content of 37.5–38.6% (Table 1). The large single copy (LSC) region in the subgenus *Hymenophyllum* was 10 kb longer than that in the subgenus *Mecodium*, due to inverted repeat (IR) expansion of the LSC region (Fig. 2). The length difference of the small single copy (SSC) region between the two subgenera mainly resulted from the insertions at the *rpl32-trnP* and *ndhA* introns in the subgenus *Mecodium*. The IR-SSC boundary was slightly different among species but positioned near the 5ʹ end of *ndhF*. There were 85 coding genes, 8 rRNA genes, 33 tRNA genes, and one pseudogene (*trnL-CAA*) in the subgenus *Hymenophyllum*. However, three species in the subgenus *Mecodium* had one more *rps7*, *ndhB* and *trnV* due to IR expansion. Gene order remained stable among species in the genus *Hymenophyllum* except the IR expansion of the subgenus *Mecodium*.

**Table 1:** Summary of plastome sequences of *Hymenophyllum.*

Subgenus	Species	Total length (bp)	LSC (bp)	SSC (bp)	IR (bp)	GC content (%)	Coverage depth (X)
*Hymenophyllum*	*H. barbatum*	144,112	102,259	20,789	10,532	37.6	227.9
	*H. holochilum* ^a^	142,214	99,299	20,765	11,075	37.6	
*Mecodium*	*H. coreanum*	157,967	89,616	21,277	23,537	38.2	367.3
	*H. polyanthos*	160,865	89,811	21,290	24,882	38.6	313.7
	*H. wrightii*	149,309	90,003	21,266	19,020	37.5	325.3

^a^Downloaded from Kuo et al. (2018).

### Length variation hotspots in *Hymenophyllum* plastomes

The intergenic spacer (IGS) between *trnV* and *rrn16* was the most variable region in length (Fig. 2). Because coverage depths between three plant genomes in a NGS data were significantly different^41^, stable read depths throughout the plastomes in this study confirmed that these length variations did not result from mitochondrial plastome sequences or nuclear plastome sequences.

In subgenus *Hymenophyllum*, *trnV-rrn16* of *H. holochilum* was 2387 bp in length and mostly belonged to the IR region. However, that of *H. barbatum* was expanded 3.4 kb more than *H. holochilum* and two-thirds of this region was part of the LSC region (Supplementary Table 1). In the subgenus *Mecodium*, *trnV-rrn16* of *H. wrightii* was 2190 bp in length and 5.7 kb and 4.4 kb expansions were confirmed at the plastomes of *H. polyanthos* and *H. coreanum,* respectively. In total, 20 ORFs having more than 100 amino acid (aa) sequences in length were also found in this length-variable region of five *Hymenophyllum* species (Supplementary Fig. 1). These ORFs ranged from 101 aa to 1274 aa in length and blastp search showed that half of them had similar amino acid sequences to ORFs that were embedded in the plastome of *Mankyua chejuense*^3^ (Table 2). Based on the DNA sequences, *trnV-rrn16* of five *Hymenophyllum* species contained at least a part of MORFFO. In addition, *H. wrightii* and *H. holochilum* contained a part of MORFFO at *trnE-trnG* and *rrn16-ycf2*, respectively.

**Table 2:** Blastp results for ORFs within *trnV-rrn16* in *Hymenophyllum* plastomes.

Query sequence			Blastp result					
Sequence Name	Name	Length (aa^a^)	Organism	Gene	Query cover (%)	E-value	Identity	Accession
*H. holochilum*	ORF_H1	103	*Pinus koraiensis*	ORF46h	20	3.40E-02	85.71	YP_001152247.1
	ORF_H2	363	*Mankyua chejuense*	ORF295	73	3.00E-76	46.99	YP_005352949.1
			*Roya anglica*	hypothetical protein	44	3.00E-07	26.95	YP_009033761.1
	ORF_H3	150	–^b^					
*H. barbatum*	ORF_B1	1,274	*Mankyua chejuense*	ORF531	41	4.00E-130	42.45	ADZ47985.2
			*Mankyua chejuense*	ORF295	22	3.00E-84	48.12	YP_005352949.1
			*Beggiatoa leptomitoformis*	AL038_02335	33	2.00E-24	28.01	ALG66761.1
	ORF_B2	101	–					
	ORF_B3	280	–					
*H. wrightii*	ORF_W1	216	–					
	ORF_W2	132	–					
*H. polyanthos*	ORF_P1	664	*Mankyua chejuense*	ORF295	44	3.00E-86	48.12	YP_005352949.1
			*Mankyua chejuense*	ORF187	18	3.00E-22	41.94	YP_005352954.1
	ORF_P2	222	–					
	ORF_P3	107	–					
	ORF_P4	147	*Mankyua chejuense*	ORF531	97	9.00E-14	37.67	YP_005352953.1
			*Bacteroidetes bacterium*	hypothetical protein	59	1.50E-01	33.33	HAV23789.1
	ORF_P5	572	*Mankyua chejuense*	ORF295	52	5.00E-77	45.97	YP_005352949.1
	ORF_P6	129	*Mankyua chejuense*	ORF531	82	5.00E-14	37.04	YP_005352953.1
	ORF_P7	107	*Thiobaca trueperi*	peptide chain release factor 3	58	3.10E-01	43.75	WP_132977102.1
*H. coreanum*	ORF_C1	1,064	*Mankyua chejuense*	ORF531	47	1.00E-123	43.24	YP_005352953.1
			*Beggiatoa leptomitoformis*	hypothetical protein	46	4.00E-25	25.54	WP_083991398.1
			*Thiotrichales bacterium HS_08*	hypothetical protein	43	3.00E-18	24.7	WP_103918394.1
	ORF_C2	218	–					
	ORF_C3	359	–					
	ORF_C4	133	–					
	ORF_C5	200	–					

^a^Amino acid.

^b^No significant similarity found.

### Enlarged noncoding regions in fern plastomes

To investigate the relationship between MORFFO and inserted loci, expanded noncoding regions having a part of MORFFO in available fern plastomes (138 plastomes, Supplementary Table 2) were found using blastn search. In total, 32 loci were matched to MORFFO with at least 100 bp in length (Supplementary table 3). Among them, 22 loci (75%) were flanked by tRNA genes. The most frequent locus, including MORFFO, throughout the plastomes was *rrn16*-(*trnV-GAC*)-*rps12* in which *trnV*-GAC was generally intact in eusporangiate ferns and early-diverging leptosporangiate ferns but was deleted or pseudogenized in most core leptosporangiate ferns.

In addition, all expanded regions had palindrome sequences at both ends with a minimum stem length of 5 bp, a minimum loop length of 5 bp, and a minimum stem-loop sequence of 20 bp.

### Phylogenetic relationships of *Hymenophyllum* species

**Figure 3:**
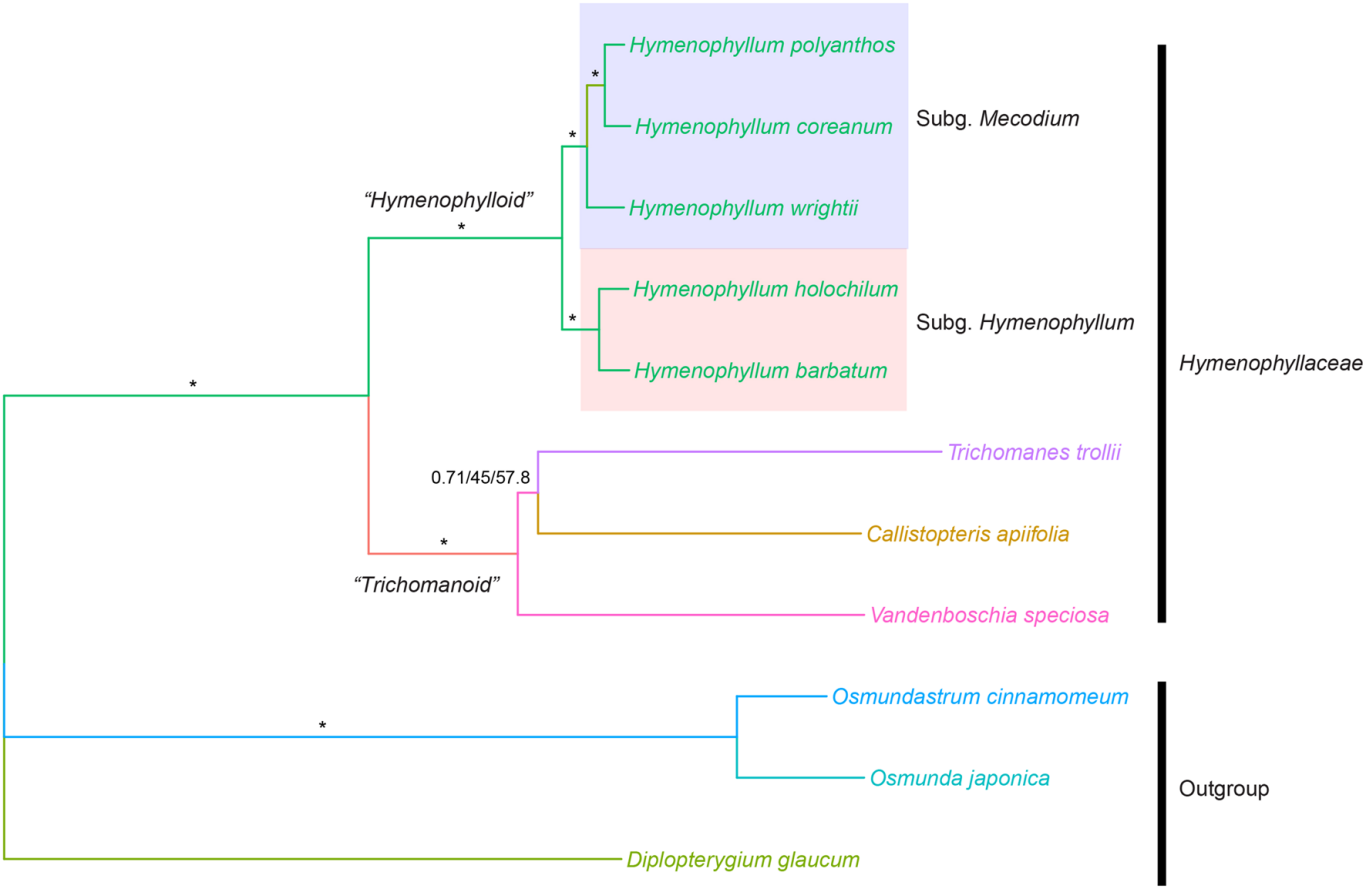
Phylogeny of the family Hymenophyllaceae using Bayesian inference with 85 genes. Numbers on the branches refer to Bayesian posterior probability/bootstrap support of ML/bootstrap support of MP. *On the branches stands for the supporting values of 1/100/100.

The concatenated alignment using 85 coding genes was 74,933 bp in length. Of these characters, 45,301 were constant, 11,129 variable characters were parsimonious uninformative, and 18,503 were parsimonious informative. Tree topologies among maximum parsimony (MP), maximum likelihood (ML) and Bayesian inference were completely identical with maximum support on all branches, with the exception of the moderate support on the clade *Callistopteris apiifolia* + *Trichomanes trollii* (Fig. 3). Hymenophyllaceae species were monophyletic and two traditional major clades, *Hymenophylloid* and *Trichomanoid*, were also strongly supported. *H. coreanum* was sister to *H. polyanthos* and they formed a clade with *H. wrightii*. As a result, two subgenera in *Hymenophyllum* were well resolved.

### Genome size variation between *H. polyanthos* and *H. coreanum*

**Figure 4:**
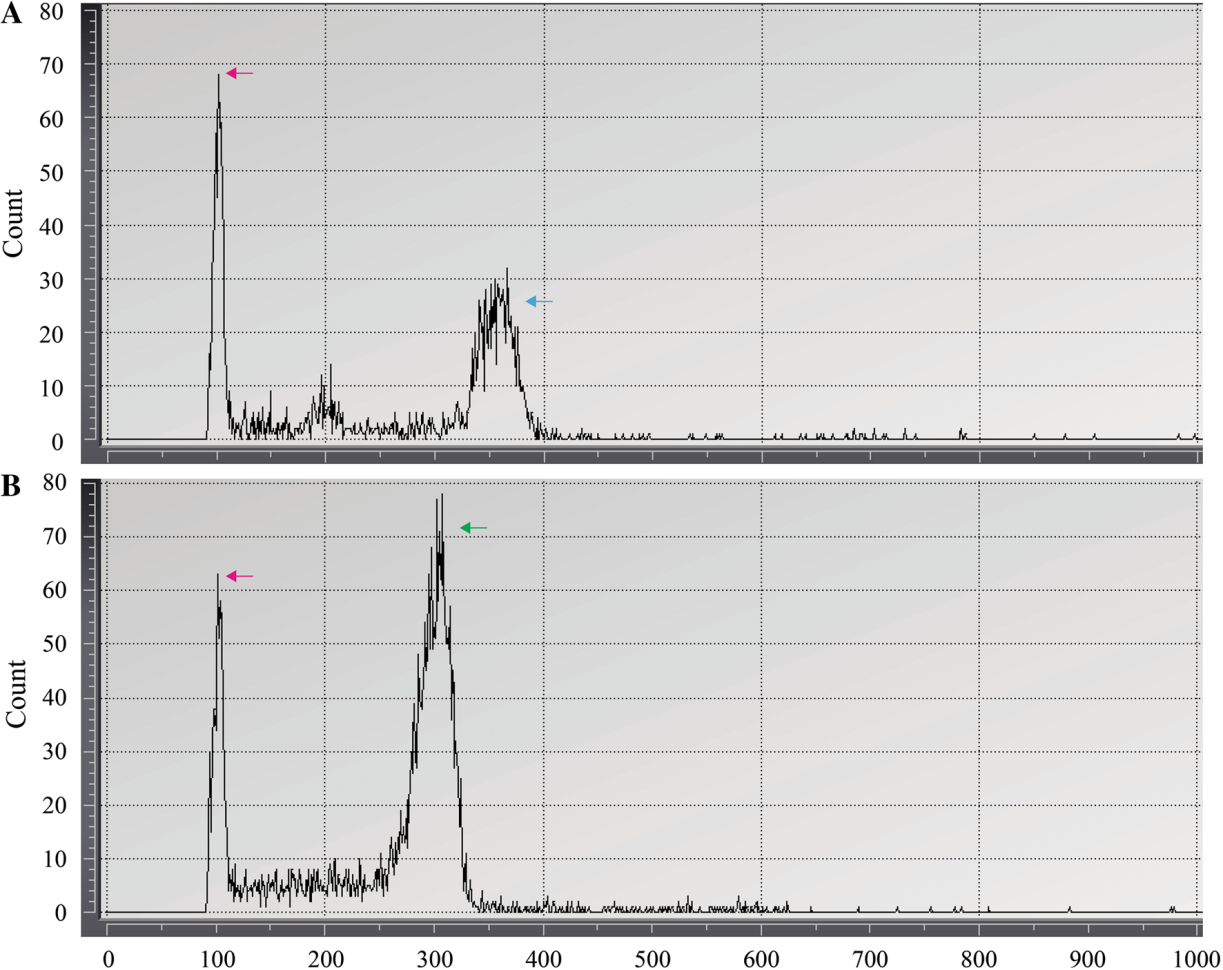
The results of the genome size measurement using the flow cytometer. (**A**) *H. polyanthos* (Blue arrow) (**B**) *H. coreanum *(Green arrow). Red arrow refers to *Nicotiana tabacum* as reference.

Because living materials of *H. coreanum* and *H. polyanthos* were very restricted in Korea, the genome size measurements for both taxa were carried out with two populations from two distant collecting sites and three populations from two distant sites, respectively. The measurement was repeated over three times for each sample. Compared to the calibration standard (1C-value of *N. tabacum* = 5.18^42^), the 1C-values of *H. polyanthos* and *H. coreanum* were 16.16 ± 0.17 and 14.85 ± 0, respectively (Fig. 4).

## Discussion

### Taxonomical position of *H. coreanum* and relationship with *H. polyanthos*

The genus *Hymenophyllum*, delimited by Morton^30^ and Iwatsuki^35^, was recently reclassified into 10 subgenera by Ebihara et al.^29^ based on morphological characters and molecular evidence^36^. Among the 10 subgenera, sensu Ebihara et al.^29^, within *Hymenophyllum*, eight subgenera have the basic chromosome number of x = 36. However, the subgenus *Mecodium* has a chromosome number of x = 28 and the subgenus *Hymenophyllum* has chromosome numbers of x = 11 to 28^29^. Because the latter two subgenera seemed to be the most recently derived taxa within the genus^36^, the ancestor of Hymenophyllales was suggested to have the chromosome number 2n = 72^43^, and it is assumed that the reduction in chromosome number is a synapomorphy of these two subgenera in the genus *Hymenophyllum*^44^.

According to Ebihara et al.^29^, the subgenus *Mecodium* consisted of *H. polyanthos* and its local derivatives. On the other hand, it was noted that *H. polyanthos* shows variation in its chromosome number^45,46^ (n = 28 Japanese taxa or 27 taxa of other country outside of Japan), and more recently, the polyphyly of this cosmopolitan species, including several regional taxa, was discovered^36,40^. Therefore, the n = 27 population of *H. polyanthos* mentioned by Iwatsuki^46^ is likely to be the result of a transition from n = 28 to n = 27.

In this study, the chromosome numbers of *H. polyanthos* and *H. coreanum* were not directly counted. However, the result of flow cytometry implied that the genome of *H. coreanum* is downsized compared with that of *H. polyanthos*. It is not clear whether *H. coreanum* has been adopted to the n = 27 population of *H. polyanthos*, mentioned by Iwatsuki^46^, based on the data at hand. However, it is clear that *H. coreanum* is distinct from *H. polyanthos* based on morphological characteristics and genome size. Therefore, *H. coreanum* should be considered as an independent species not one of the synonymous taxa of *H. polyanthos.*

**Figure 5:**
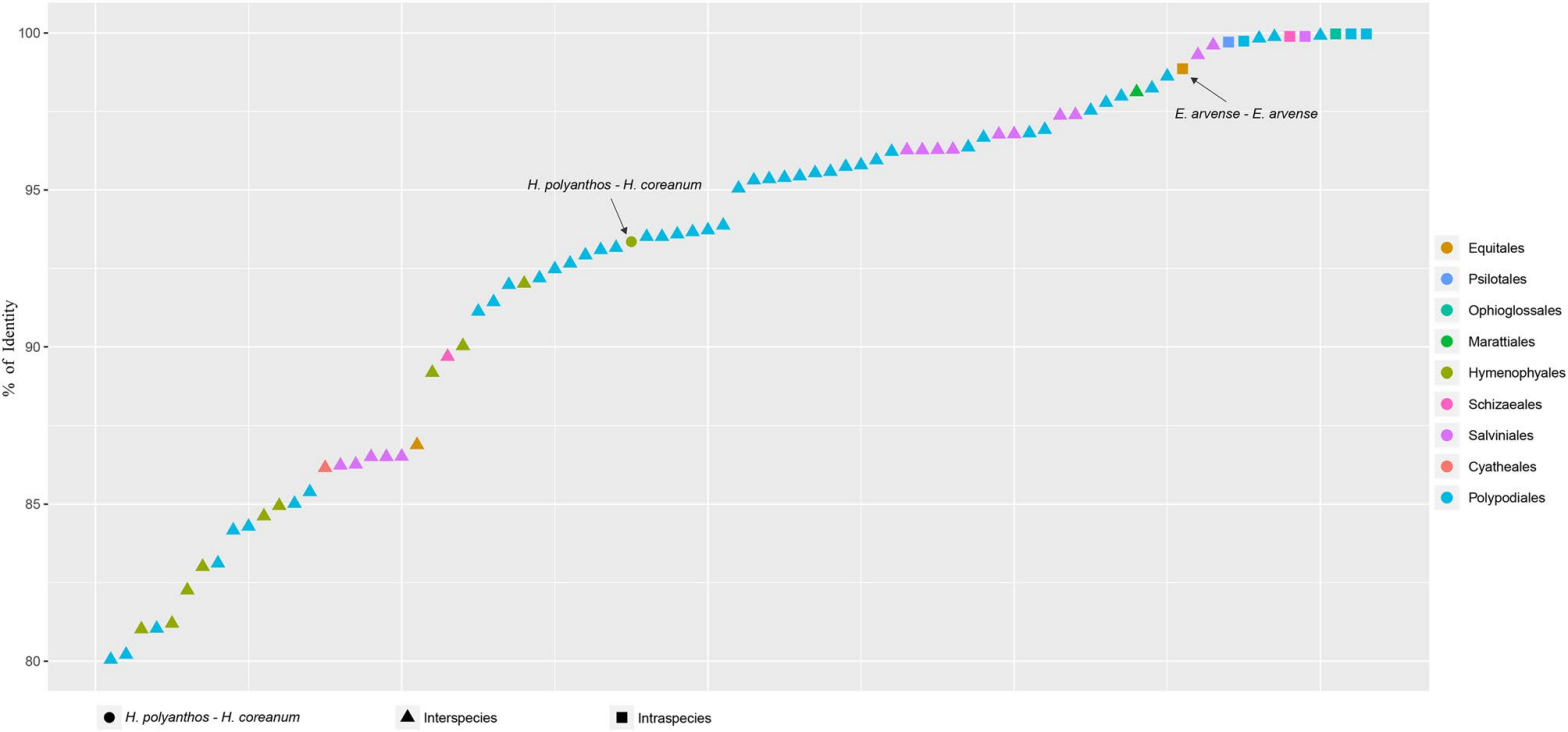
Percentage of sequence identity between two plastomes. Circle refers to the sequence identity between *H. polyanthos* and *H. coreanum*. Triangle and square refer to the sequence identity between two plastomes of interspecific level in the same genus and that of infraspecific level in the same species, respectively.

The divergence of plastome sequences between both species also strongly supports this taxonomical treatment. So far, it was rare to report more than two plastome sequences in the same species of ferns (Fig. 5 and supplementary table 4). The percentage of sequence identity between two plastomes in a species ranges from 98.86 (*Equisetum arvense*) to 99.97% (*Mankyua chejuense*). Except for the case of *E. arvense* in which the copy number variations frequently occurred^15^, sequence identities within the same species are higher than 99.7% in ferns. In contrast, the sequence identities between the plastomes of two different species in a genus, with the exception of the genera *Cyrtomium* and *Azolla*, are less than 98.7% (Fig. 5 and supplementary table 4). Sequence identity between plastomes of *H. polyanthos* and *H. coreanum* is quite low at 93.35% in the whole sequence and 98.13% even if the variation in *trnV-rrn16* IGS was ignored. Consequently, plastome sequences of these two species revealed that they are quite distinct from each other and there is no reason to consider them as an intraspecific variation.

In this study, we only investigated the Korean population of *H. coreanum* and it is not known whether the *H. coreanum* distributed in China, Taiwan and Japan^38^ also has the same genome size and plastome sequences as the Korean population. In Korea, the species mainly inhabits high mountains, however information for this species is very poor because it has previously been considered a synonymous taxon of *H. polyanthos*. Further study of this species facilitate better understanding of the speciation of the subgenus *Mecodium* because many species within the subgenus are derivatives of *H. polyanthos*, and *H. coreanum*, at least in Korea, seems to be recently diverged from *H. polyanthos*.

### MORFFO of ferns prefer stem-loop structures

In previous studies^25,47^, the physical positions of MORFFO were considered to be related to inversions or IR borders even though it is not clear whether MORFFO are a cause or a consequence of inversions. In the present study, we made a new finding that the positions of MORFFO were between palindrome sequences, especially near tRNA genes (Supplementary table 3). Transfer RNA genes are very important in structural variation in fern plastomes. For instance, inversion between *trnfM* and *trnE* is specific to moninophytes^48^ and a number of inversions occurring near tRNA genes have been found in fern plastomes^3,19,26,27^. In addition, length variations in noncoding regions of plastomes owing to tRNA gene repeats have been suggested^19,27^. Transfer RNA-related genome rearrangements are not unique characteristic of fern plastomes. Intermolecular recombination between distinct tRNA genes has been found in cereals^49^, and extensive rearrangements were also suggested due to repeats or tRNA genes in *Trachelium caeruleum*^50^. Consequently, tRNA genes in plastomes of ferns seem to be strongly associated with genome instability events which can lead to insertion of MORFFO using double-strand breaks^51^.

In bacterial genome evolution, the most transposable elements have short terminal inverted repeats^52^. The insertion of some specific sequences related to repetitive extragenic palindromic (REP) elements have been reported^53,54^. Therefore, the mobility of MORFFO within the plastome is likely to be associated to the palindrome sequences at both ends of insertion regions. Two MORFFO in distant positions in a plastome of certain species in Polypodiales^47^ and Hymenophyllales in this study also seem to be caused by the palindrome sequences.

Correctively, it is likely that the mobile elements, consisting of palindrome sequences, lead to the insertion of MORFFO in the plastomes of ferns, and genome instability caused by hairpin structures at the ends of MORFFO influence genome rearrangements on the plastome. As a result, inversions seem to frequently occur in the loci having MORFFO.

### Degradation after insertion of long DNA contig including MORFFO

So far, more than one hundred plastome sequences in ferns have been reported, and MORFFO have been found within at least one species in Marattiales, Ophioglossales, Hymenophyllales, Gleicheniales, Schizaeales, Cyatheales, and Polypodiales (Supplementary Table 2). The MORFFO status (present or absent) varies at the family level in Polypodiales^47^ or at the genus level in Ophioglossaceae, Schizaeaceae, Blechnaceae, Dryopteridaceae, Lomariopsidaceae, Polypodiaceae, Pteridaceae, and Thelypteridaceae. In addition, the inserted region of MORFFO also differs in certain genera^25^ (i.e., *Asplenium*, *Cheilanthes*, *Myriopteris*). It is uncertain exactly when MORFFO were inserted in the fern plastomes. However, if MORFFO were independent events at different lineages of ferns, it is difficult to explain (1) why expanded regions, including MORFFO, have high sequence similarities throughout the fern lineages^25^ and (2) how MORFFO were frequently transferred into the highly conserved genome, in terms of gene transfer^55^. In addition, MORFFO-like sequences found in cyanobacteria or green algae imply that MORFFO come from a single origin that was started from nuclear genomes after endosymbiotic or horizontal gene transfer^3,25^.

Even though MORFFO are supposed to be under selective pressure^25^, they are not assumed to play a vital role for fern plastomes because there was no vestige of MORFFO in various fern families (Supplementary Table 2). It is not surprising to degrade unnecessary genes in a plastome of land plants^56,57^. Especially, the degradation of *ndh* genes in Orchidaceae showed how easy it is to delete genes in plastomes among closely related taxa^58^. MORFFO in the plastome of *Mankyua* and that in the mitochondrial genome of *Helminthostachys*^3^ also suggest MORFFO degradation through intracellular gene transfer.

In Hymenophyllaceae, the length of *rrn16-trnV* of *H. wrightii* is slightly expanded similar to previously reported plastomes^20,59^, except *Vandenboschia*^60^, owing to MORFFO. Interestingly, *rrn16-trnV* is almost tripled in *H. barbatum* and *H. coreanum* or quadrupled in *H. polyanthos* compared with *H. wrightii* (Fig. 2)*.* As mentioned earlier, MORFFO seems to have existed in the ancestor of *Hymenophyllum*, the length variation of *rrn16-trnV* in *Hymenophyllum* is able to be interpreted into a consequence of degradation.

Similar to the plastomes in Ophioglossaceae and *Hymenophyllum*, MORFFO survived or disappeared during the evolution of fern plastomes. Partial sequences of MORFFO in most core leptosporangiate ferns except Pteridaceae^25^ are consistent with the degradation hypothesis. MORFFO-lacking lineages may come from an ancestor that has undergone an independent MORFFO degradation event. However, it is not clear why MORFFO remain in relatively recently diverged families in the fern phylogeny^17^, Pteridaceae of Polypodiales^25^, if they do not have any function. One hypothesis is that *morffo1*, *morffo2*, and *morffo3*, which are found in Pteridaceae, are not intact genes but partial genes of a large ORF. Robison et al.^25^ described that these three ORFs cluster immediately adjacent to one another when they are present, and *morffo1* and *morffo2* often form a larger ORF. ORF_B1 in *H. barbatum* is highly similar to *morffo1* and *morffo2* consistent with their observation. If insertion of MORFFO contig was the consequence of genomic instability by stem-loop structures and then rearrangements including insertion/deletion and inversion were caused by genomic instability at the inserted region, a larger ORF can be fragmented and shuffling of smaller fragments can make novel chimeric ORFs. Actually, these novel chimeric ORFs have been reported in the mitochondrial genomes of land plants^61,62^.

Plastomes of eusporangiate and basal leptosporangiate ferns will shed light on the prototype of extant MORFFO, and this information will lead us to understand the evolution of MORFFO in fern plastomes.

## Materials and Methods

### Plant materials and DNA extraction

Four species, *H. barbatum* (CBNU-2018-0021), *H. polyanthos* (CBNU-2018-0187), *H. wrightii* (CBNU-2018-0330), and *H. coreanum* (CBNU-2018-0190), were sampled at Cheju Island and Jirisan in Korea. All specimens and living materials were deposited at the herbarium and greenhouse of Chungbuk National University. Using a fresh leaf, genomic DNAs were extracted by means of DNeasy Plant Mini Kit (Qiagen, Inc.) following the protocol proposed by the manufacturer.

#### Sequencing and assembling

Four high-quality DNAs were sequenced by Illimina HSeq X Ten. The raw reads were trimmed by trimmomatic 0.36^63^ with the options of leading:10, trailing:10, slidingwindow:4:20, and minlen:50. The assembly method was identical to Kim et al.^64^ using *Hymenophyllum hollochilum* (NC_039753) as a reference sequence.

#### Gene annotation

Genes in four plastome sequences were annotated compared with the genes of *H. hollochilum* using Geneious 10.2.6. Transfer RNA genes were verified using tRNAscan-SE^65^ and ORFs with > 303 bp in length were also annotated using Geneious 10.2.6.

#### Analyses of ORFs in *Hymenophyllum* and expanded noncoding regions in fern plastomes

To investigate the ORFs located in the *rrn16-trnV* IGS having high length variation in the genus, we extracted that the region from four plastome sequences. First, *rrn16-trnV* IGS was compared to other nucleotides using blastn^66^ to find out the homologous sequences. Thereafter, ORFs were searched using blastp to confirm the origin of these ORFs. To explore the physical positions of MORFFO, 126 plastome sequences were downloaded from NCBI and eight plastome sequences were received from Lehtonen and Cárdenas^47^ (Supplementary Table 2).

#### Phylogenetic relationships among four *Hymenophyllum* species

A total of 85 coding genes, except duplicated genes, were extracted from eight Hymenophyllaceae species and two Osmundales and one Gleicheniales species. To obtain the best partitioning scheme for each gene position in a single concatenated sequence, ModelFinder^67^ and PartitionFinder^68^ were used for ML analysis and BI analysis, respectively. MP and ML analyses were conducted using PAUP^69^ and IQ-TREE^70^ with 1000 bootstrap replications. BI analysis was inferred by Mrbayes^71^ under selected models in PartitionFinder with ngen = 1,000,000, samplefreq = 500, burninfrac = 0.25.

#### Estimation of DNA amounts

The genome size of *H. polyanthos* and *H. coreanum* was measured using living materials. For *H. polyanthos*, three populations were collected from Mt. Jiri-san in mainland of Korean peninsula and Mt. Halla-san of Jeju Island located at the most southern region of Korea. Two populations of *H. coreanum* were also collected from Mt. Jiri-san and Mt. Gariwang-san of southern and northern part of Korea, respectively. For a calibration standard, we grew *Nicotiana tabacum* in a greenhouse for weeks and used only 1 × 1 cm^2^ of young leaf. The reference and two samples were separately chopped with 500 μl of Nuclei extraction buffer in CyStain UV Precise P kit (Sysmex Partec, Germany) in a Petri dish on ice. The extraction time for samples was extended for eight minutes because the numbers of events less than that time were very low to visualize the graph. The debris was filtered using non-sterile CellTrics filter Green 30 μm (Sysmex Partec, Germany) and nuclei were stained by Staining Buffer in CyStain UV Precise P kit (Sysmex Partec, Germany). Particles of each sample were measured using CyFlow Cube 6 flow cytometer (Sysmex Partec, Germany).

## Supplementary information

Supplementary file1 (DOCX 3060 kb)

## Data Availability

The complete sequence data used in the current study are available in the NCBI GenBank repository. All data generated or analyzed during this study are included in this published article and its Supplementary Information files.
