# Supplementary material for: The dynamic evolution of mobile open reading frames in plastomes of Hymenophyllum Sm. and new insight on Hymenophyllum coreanum Nakai
Source: Sci Rep. 2020 Jul 6;10:11059. doi: 10.1038/s41598-020-68000-7 (PMC7338519; doi:10.1038/s41598-020-68000-7)
Supplement: Supplementary file 1 — Supplementary file1 (DOCX 3060 kb) [file 41598_2020_68000_MOESM1_ESM.docx]

Supplementary Material

**The dynamic evolution of mobile open reading frames in plastomes of *Hymenophyllum* Sm. and new insight on *Hymenophyllum coreanum*  Nakai**

Hyoung Tae Kim^1^, Jung Sung Kim^2^*

1 Institute of Agricultural Science and Technology, Chungbuk National University, Chungbuk, 28644, Korea

2 Department of Forest Science, Chungbuk National University, Chungbuk, 28644, Korea

**Corresponding author**

**Jung Sung Kim**

**e-mail:** [**jungsung@chungbuk.ac.kr**](mailto:jungsung@chungbuk.ac.kr)

**Tel: +82-43-261-2535**

Supplementary table 1. Intergenic spacer between *trnV* and *rrn16* in *Hymenophyllum* plastomes.

| Taxon | Length (bp) | Coverage depth (X) | ORFs |
| --- | --- | --- | --- |
| *H. holochilum* | 2,387 |  | ORF_H1, ORF_H2, ORF_H3 |
| *H. barbatum* | 5,727 | 212.5 | ORF_B1, ORF_B2, ORF_B3, ORF_B4 |
| *H. coreanum* | 6,625 | 307.5 | ORF_C1, ORF_C2, ORF_C3, ORF_C4, ORF_C5 |
| *H. polyanthos* | 7,908 | 245.9 | ORF_P1, ORF_P2, ORF_P3, ORF_P4, ORF_P5, ORF_P6, ORF_P7, ORF_P8 |
| *H. wrightii* | 2,190 | 267.7 | ORF_W1, ORF_W2, ORF_W3 |

Supplementary table 2. List of 138 plastomes to investigate the relationship between MORFFO and inserted loci.

| Taxon | Accession | Order | Family | MORFFO ^a^ |
| --- | --- | --- | --- | --- |
| *Equisetum arvense* 1 | NC_014699 | Equisetales | Equisetaceae | - |
| *Equisetum arvense* 2 | JN968380 | Equisetales | Equisetaceae | - |
| *Equisetum hyemale* | NC_020146 | Equisetales | Equisetaceae | - |
| *Angiopteris angustifolia* | NC_026300 | Marattiales | Marattiaceae | o |
| *Angiopteris evecta* | NC_008829 | Marattiales | Marattiaceae | o |
| *Botrychium* sp. | KM817789.2 | Ophioglossales | Ophioglossaceae | - |
| *Helminthostachys zeylanica* | KM817788 | Ophioglossales | Ophioglossaceae | o |
| *Mankyua chejuensis* 1 | NC_017006 | Ophioglossales | Ophioglossaceae | o |
| *Mankyua chejuensis* 2 | KP205433 | Ophioglossales | Ophioglossaceae | o |
| *Ophioglossum californicum* | NC_020147 | Ophioglossales | Ophioglossaceae | o |
| *Psilotum nudum* 1 | NC_003386 | Psilotales | Psilotaceae | - |
| *Psilotum nudum* 2 | KC117179 | Psilotales | Psilotaceae | - |
| *Tmesipteris elongata* | KJ569699 | Psilotales | Psilotaceae | - |
| *Osmunda japonica* | MK554796.1 | Osmundales | Osmundaceae | - |
| *Osmundastrum cinnamomeum* | NC_024157 | Osmundales | Osmundaceae | - |
| *Callistopteris apiifolia* | MH265125.1 | Hymenophyllales | Hymenophyllaceae | o |
| *Hymenophyllum barbatum* |  | Hymenophyllales | Hymenophyllaceae | o |
| *Hymenophyllum coreanum* |  | Hymenophyllales | Hymenophyllaceae | o |
| *Hymenophyllum holochilum* | NC_039753.1 | Hymenophyllales | Hymenophyllaceae | o |
| *Hymenophyllum polyanthos* |  | Hymenophyllales | Hymenophyllaceae | o |
| *Hymenophyllum wrightii* |  | Hymenophyllales | Hymenophyllaceae | o |
| *Trichomanes trollii* | NC_041122.1 | Hymenophyllales | Hymenophyllaceae | o |
| *Vandenboschia speciosa* | NC_041000.1 | Hymenophyllales | Hymenophyllaceae | - |
| *Diplopterygium glaucum* | NC_024158 | Gleicheniales | Gleicheniaceae | o |
| *Lygodium japonicum* 1 | NC_022136 | Schizaeales | Lygodiaceae | - |
| *Lygodium japonicum* 2 | KF225593 | Schizaeales | Lygodiaceae | - |
| *Lygodium microphyllum* | NC_039378.1 | Schizaeales | Lygodiaceae | o |
| *Actinostachys pennula* | KU764518 | Schizaeales | Schizaeaceae | o |
| *Schizaea elegans* | NC_035807 | Schizaeales | Schizaeaceae | - |
| *Schizaea pectinata* | NC_035808 | Schizaeales | Schizaeaceae | o |
| *Marsilea crenata* | NC_022137 | Salviniales | Marsileaceae | - |
| *Pilularia americana* | KY863504.1 | Salviniales | Marsileaceae | - |
| *Azolla caroliniana* | MF177092 | Salviniales | Salviniaceae | - |
| *Azolla filiculoides* | MF177094 | Salviniales | Salviniaceae | - |
| *Azolla mexicana* | MF177088 | Salviniales | Salviniaceae | - |
| *Azolla microphylla* | MF177089 | Salviniales | Salviniaceae | - |
| *Azolla nilotica* | MF177090 | Salviniales | Salviniaceae | - |
| *Azolla rubra* | MF177091 | Salviniales | Salviniaceae | - |
| *Salvinia cucullata* | MF177095 | Salviniales | Salviniaceae | - |
| *Cibotium barometz* | NC_037893.1 | Cyatheales | Cibotiaceae | o |
| *Alsophila podophylla* | NC_038150.1 | Cyatheales | Cyatheaceae | o |
| *Alsophila spinulosa* | NC_012818 | Cyatheales | Cyatheaceae | o |
| *Dicksonia squarrosa* | KJ569698 | Cyatheales | Dicksoniaceae | o |
| *Plagiogyria glauca* | KP136831 | Cyatheales | Plagiogyriaceae | o |
| *Asplenium pekinense* | NC_035837 | Polypodiales | Aspleniaceae | o |
| *Asplenium prolongatum* | NC_035838 | Polypodiales | Aspleniaceae | o |
| *Hymenasplenium unilaterale* | NC_035856 | Polypodiales | Aspleniaceae | o |
| *Athyrium anisopterum* | NC_035738 | Polypodiales | Athyriaceae | - |
| *Athyrium opacum* | NC_035841 | Polypodiales | Athyriaceae | - |
| *Athyrium sheareri* | NC_035836 | Polypodiales | Athyriaceae | - |
| *Athyrium sinense* | NC_035839 | Polypodiales | Athyriaceae | - |
| *Deparia lancea* 1 | NC_035844 | Polypodiales | Athyriaceae | - |
| *Deparia lancea* 2 | MH124235.1 | Polypodiales | Athyriaceae | - |
| *Deparia pycnosora* | NC_035845 | Polypodiales | Athyriaceae | - |
| *Deparia viridifrons* | NC_035846 | Polypodiales | Athyriaceae | - |
| *Diplazium bellum* | NC_035849 | Polypodiales | Athyriaceae | - |
| *Diplazium dilatatum* | NC_035850 | Polypodiales | Athyriaceae | - |
| *Diplazium dushanense* | NC_035851 | Polypodiales | Athyriaceae | - |
| *Diplazium striatum* | NC_035852 | Polypodiales | Athyriaceae | - |
| *Diplazium unilobum* | NC_035853 | Polypodiales | Athyriaceae | - |
| *Austroblechnum melanocaulon* | NC_035840 | Polypodiales | Blechnaceae | - |
| *Woodwardia unigemmata* | NC_028543 | Polypodiales | Blechnaceae | o |
| *Cystopteris chinensis* | NC_035843 | Polypodiales | Cystopteridaceae | - |
| *Cystopteris protrusa* | KP136830 | Polypodiales | Cystopteridaceae | - |
| *Davallia fejeensis ^b^* |  | Polypodiales | Davalliaceae | o |
| *Histiopteris incisa* | NC_040220.1 | Polypodiales | Dennstaedtiaceae | o |
| *Pteridium aquilinum* subsp*. aquilinum* | NC_014348 | Polypodiales | Dennstaedtiaceae | o |
| *Diplaziopsis cavaleriana* | NC_035847 | Polypodiales | Diplaziopsidaceae | - |
| *Diplaziopsis javanica* | NC_035848 | Polypodiales | Diplaziopsidaceae | - |
| *Homalosorus pycnocarpos* | NC_035855 | Polypodiales | Diplaziopsidaceae | - |
| *Cyrtomium devexiscapulae* | NC_028542 | Polypodiales | Dryopteridaceae | - |
| *Cyrtomium falcatum* | NC_028705 | Polypodiales | Dryopteridaceae | - |
| *Cyrtomium fortunei* | NC_037510.1 | Polypodiales | Dryopteridaceae | - |
| *Dryopteris blanfordii* | LT827127.1 | Polypodiales | Dryopteridaceae | - |
| *Dryopteris crassirhizoma* | MK554795.1 | Polypodiales | Dryopteridaceae | - |
| *Dryopteris decipiens* | NC_035854.1 | Polypodiales | Dryopteridaceae | - |
| *Dryopteris filix-mas* | LT618774.1 | Polypodiales | Dryopteridaceae | - |
| *Dryopteris fragrans* | KX418656 | Polypodiales | Dryopteridaceae | o |
| *Dryopteris villarii* | LT905144.1 | Polypodiales | Dryopteridaceae | - |
| *Hypodematium crenatum* | NC_035857 | Polypodiales | Hypodematiaceae | - |
| *Lindsaea linearis ^b^* |  | Polypodiales | Lindsaeaceae | - |
| *Odontosoria chinensis* | MG913608.1 | Polypodiales | Lindsaeaceae | o |
| *Lomariopsis japurensis ^b^* |  | Polypodiales | Lomariopsidaceae | o |
| *Nephrolepis biserrata ^b^* |  | Polypodiales | Lomariopsidaceae | - |
| *Oleandra articulata ^b^* |  | Polypodiales | Oleandraceae | o |
| *Matteuccia struthiopteris* | NC_035859.1 | Polypodiales | Onocleaceae | - |
| *Onoclea sensibilis* | NC_035860.1 | Polypodiales | Onocleaceae | - |
| *Aglaomorpha fortunei* | KY075853.1 | Polypodiales | Polypodiaceae | o |
| *Goniophlebium niponicum* | NC_040221.1 | Polypodiales | Polypodiaceae | - |
| *Lepisorus clathratus* | NC_035739 | Polypodiales | Polypodiaceae | o |
| *Polypodium glycyrrhiza* | KP136832 | Polypodiales | Polypodiaceae | o |
| *Pyrrosia bonii* | NC_040226.1 | Polypodiales | Polypodiaceae | o |
| *Leptochilus hemionitideus* | NC_040177.1 | Polypodiales | Polypodiineae | o |
| *Pecluma dulce ^b^* |  | Polypodiales | Polypodiineae | o |
| *Adiantum aleuticum* | NC_040209.1 | Polypodiales | Pteridaceae | o |
| *Adiantum capillus-veneris* | NC_004766 | Polypodiales | Pteridaceae | - |
| *Adiantum hispidulum* | LT615217.1 | Polypodiales | Pteridaceae | - |
| *Adiantum shastense* | NC_037478.1 | Polypodiales | Pteridaceae | - |
| *Anogramma chaerophylla* | NC_040210.1 | Polypodiales | Pteridaceae | o |
| *Antrophyum semicostatum* | NC_040176.1 | Polypodiales | Pteridaceae | o |
| *Bommeria hispida* | NC_040206.1 | Polypodiales | Pteridaceae | o |
| *Calciphilopteris ludens* | NC_040214.1 | Polypodiales | Pteridaceae | o |
| *Ceratopteris cornuta* | NC_040204.1 | Polypodiales | Pteridaceae | o |
| *Ceratopteris richardii* | KM052729 | Polypodiales | Pteridaceae | o |
| *Cheilanthes lindheimeri* | NC_014592 | Polypodiales | Pteridaceae | o |
| *Cheilanthes micropteris* | NC_040174.1 | Polypodiales | Pteridaceae | o |
| *Cryptogramma acrostichoides* | NC_040211.1 | Polypodiales | Pteridaceae | o |
| *Haplopteris elongata* | NC_040215.1 | Polypodiales | Pteridaceae | o |
| *Hemionitis subcordata* | NC_040173.1 | Polypodiales | Pteridaceae | o |
| *Jamesonia brasiliensis* | MH173077.1 | Polypodiales | Pteridaceae | o |
| *Llavea cordifolia* | NC_040216.1 | Polypodiales | Pteridaceae | - |
| *Myriopteris covillei* | NC_039724.1 | Polypodiales | Pteridaceae | o |
| *Myriopteris scabra* | NC_040213.1 | Polypodiales | Pteridaceae | o |
| *Notholaena standleyi* | NC_040203.1 | Polypodiales | Pteridaceae | o |
| *Onychium japonicum* | NC_040205.1 | Polypodiales | Pteridaceae | - |
| *Pellaea truncata* | NC_040202.1 | Polypodiales | Pteridaceae | - |
| *Pentagramma triangularis* | NC_040171.1 | Polypodiales | Pteridaceae | o |
| *Pityrogramma trifoliata* | NC_040207.1 | Polypodiales | Pteridaceae | o |
| *Pteris vittata* 1 | NC_040212.1 | Polypodiales | Pteridaceae | o |
| *Pteris vittata* 2 | MH500228.1 | Polypodiales | Pteridaceae | o |
| *Scoliosorus ensiformis* | NC_040218.1 | Polypodiales | Pteridaceae | - |
| *Tryonia myriophylla* | NC_040208.1 | Polypodiales | Pteridaceae | o |
| *Vaginularia trichoidea* | NC_040175.1 | Polypodiales | Pteridaceae | o |
| *Vittaria appalachiana* | NC_040219.1 | Polypodiales | Pteridaceae | o |
| *Vittaria graminifolia* | NC_040217.1 | Polypodiales | Pteridaceae | o |
| *Rhachidosorus consimilis* | NC_035862 | Polypodiales | Rhachidosoraceae | o |
| *Saccoloma inaequale ^b^* |  | Polypodiales | Saccolomataceae | o |
| *Tectaria panamensis ^b^* |  | Polypodiales | Tectariaceae | o |
| *Ampelopteris prolifera* | NC_035835.1 | Polypodiales | Thelypteridaceae | - |
| *Christella appendiculata* | NC_035842.1 | Polypodiales | Thelypteridaceae | - |
| *Macrothelypteris torresiana* 1 | NC_035858 | Polypodiales | Thelypteridaceae | - |
| *Macrothelypteris torresiana* 2 | MH500230.1 | Polypodiales | Thelypteridaceae | - |
| *Mesopteris tonkinensis* | NC_041428.1 | Polypodiales | Thelypteridaceae | o |
| *Pseudophegopteris aurita* | KY427355 | Polypodiales | Thelypteridaceae | - |
| *Stegnogramma sagittifolia* | NC_035863 | Polypodiales | Thelypteridaceae | - |
| *Thelypteris aurita* | NC_035861 | Polypodiales | Thelypteridaceae | - |
| *Woodsia macrochlaena* | NC_035864 | Polypodiales | Woodsiaceae | - |
| *Woodsia polystichoides* | NC_035865 | Polypodiales | Woodsiaceae | - |

^a^ : - (absent), o (present)

^b^ : Lehtonen and Cárdenas (2019)

Supplementary table 3. Expanded noncoding regions having MORFFO in 138 fern plastomes

| Region | tRNA* | Taxon | Accession |
| --- | --- | --- | --- |
| *accD - psaI* | - | *Lomariopsis japurensis* |  |
| *accD - rbcL* | *trnR-UCG* | *Aglaomorpha fortunei* | KY075853.1 |
| *accD - trnR* | *trnR-UCG* | *Anogramma chaerophylla* | NC_040210.1 |
|  |  | *Pecluma dulce* |  |
| *ccsA - trnN* | *trnN-GUU* | *Actinostachys pennula* | KU764518.1 |
| *ndhB - trnL* | *trnL-CAA* | *Angiopteris angustifolia* | NC_026300 |
|  |  | *Angiopteris evecta* | NC_008829 |
| *ndhC - trnV* | *trnV-UAC* | *Ceratopteris cornuta* | NC_040204.1 |
|  |  | *Ceratopteris richardii* | KM052729 |
|  |  | *Cheilanthes micropteris* | NC_040174.1 |
|  |  | *Histiopteris incisa* | NC_040220.1 |
|  |  | *Pteridium aquilinum* subsp*. aquilinum* | NC_014348.1 |
| *ndhF - ycf2* | *-* | *Davallia fejeensis* |  |
| *ndhJ - trnF* | *trnF-GAA* | *Helminthostachys zeylanica* | KM817788.2 |
| *petA - psbJ* | *-* | *Pyrrosia bonii* | NC_040226.1 |
| *petL - psbE* | *-* | *Pentagramma triangularis* | NC_040171.1 |
| *petN - psbM* | *-* | *Odontosoria chinensis* | MG913608.1 |
|  |  | *Pteris vittata* | NC_040212.1 |
|  |  | *Pteris vittata* | MH500228.1 |
| *psaC - rps15* |  | *Schizaea pectinata* | NC_035808 |
| *psbM - trnD* | *trnD-GUC* | *Plagiogyria glauca* | KP136831.1 |
| *psbM - trnE* | *trnE-UUC* | *Odontosoria chinensis* | MG913608.1 |
| *rpoB - trnD* | *trnD-GUC* | *Antrophyum semicostatum* | NC_040176.1 |
|  |  | *Vaginularia trichoidea* | NC_040175.1 |
|  |  | *Vittaria appalachiana* | NC_040219.1 |
|  |  | *Vittaria graminifolia* | NC_040217.1 |
| *rps12 - trnV* | *trnV-GAC* | *Diplopterygium glaucum* | NC_024158.1 |
| *rps15 - ycf1* | *-* | *Bommeria hispida* | NC_040206.1 |
|  |  | *Leptochilus hemionitideus* | NC_040177.1 |
|  |  | *Myriopteris scabra* | NC_040213.1 |
| *rps4 - trnL* | *trnL-UAA* | *Hymenasplenium unilaterale* | NC_035856.1 |
|  |  | *Mankyua chejuensis* | NC_017006.1 |
| *rps12 - rrn5* | *-* | *Antrophyum semicostatum* | NC_040176.1 |
|  |  | *Haplopteris elongata* | NC_040215.1 |
| *rrn16 - rps12* | *trnV-GAC* | *Adiantum aleuticum* | NC_040209.1 |
|  |  | *Alsophila podophylla* | NC_038150.1 |
|  |  | *Asplenium pekinense* | NC_035837 |
|  |  | *Calciphilopteris ludens* | NC_040214.1 |
|  |  | *Cibotium barometz* | NC_037893.1 |
|  |  | *Dicksonia squarrosa* | KJ569698.1 |
|  |  | *Dryopteris fragrans* | KX418656 |
|  |  | *Hemionitis subcordata* | NC_040173.1 |
|  |  | *Histiopteris incisa* | NC_040220.1 |
|  |  | *Hymenasplenium unilaterale* | NC_035856.1 |
|  |  | *Lepisorus clathratus* | NC_035739.1 |
|  |  | *Lygodium microphyllum* | NC_039378.1 |
|  |  | *Myriopteris scabra* | NC_040213.1 |
|  |  | *Notholaena standleyi* | NC_040203.1 |
|  |  | *Odontosoria chinensis* | MG913608.1 |
|  |  | *Polypodium glycyrrhiza* | KP136832.1 |
|  |  | *Pteris vittata* | NC_040212.1 |
|  |  | *Pteris vittata* | MH500228.1 |
|  |  | *Pyrrosia bonii* | NC_040226.1 |
|  |  | *Rhachidosorus consimilis* | NC_035862.1 |
|  |  | *Tectaria panamensis* |  |
|  |  | *Woodwardia unigemmata* | NC_028543.1 |
| *rrn16 - trnV* | *trnV-GAC* | *Callistopteris apiifolia* | MH265125.1 |
|  |  | *Hymenophyllum barbatum* | #N/A |
|  |  | *Hymenophyllum coreanum* | #N/A |
|  |  | *Hymenophyllum holochilum* | NC_039753.1 |
|  |  | *Hymenophyllum polyanthos* | #N/A |
|  |  | *Hymenophyllum wrightii* | #N/A |
|  |  | *Trichomanes trollii* | NC_041122.1 |
| *rrn16 - ycf2* | *-* | *Hymenophyllum holochilum* | NC_039753.1 |
| *trnC - trnG* | *trnC-GCA, trnG-GCC* | *Alsophila spinulosa* | NC_012818.1 |
| *trnD - trnY* | *trnD-GUC, trnY-GUA* | *Tryonia myriophylla* | NC_040208.1 |
| *trnE - trnG* | *trnE-UUC, trnG-GCC* | *Hymenophyllum wrightii* |  |
| *trnfM - trnT* | *trnfM-CAU, trnT-GGU* | *Ophioglossum californicum* | NC_020147.1 |
| *trnH – ycf2* | *trnH-GUG* | *Saccoloma inaequale* |  |
| *trnM - trnV* | *trnM-CAU, trnV-UAC* | *Pityrogramma trifoliata* | NC_040207.1 |
| *trnN - ycf2* | *trnN-GUU* | *Asplenium prolongatum* | NC_035838.1 |
|  |  | *Cheilanthes lindheimeri* | NC_014592.1 |
|  |  | *Jamesonia brasiliensis* | MH173077.1 |
|  |  | *Myriopteris covillei* | NC_039724.1 |
| *trnR - trnT* | *trnR-ACG, trnT-UGU* | *Mesopteris tonkinensis* | NC_041428.1 |
| *trnT_intron* | *trnT-UGU* | *Oleandra articulata* |  |
|  |  | *Saccoloma inaequale* |  |
| *trnV intron* | *trnV-UAC* | *Cryptogramma acrostichoides* | NC_040211.1 |

* tRNA genes are flanked by expanded region

Supplementary table 4. Percent of identity between two plastomes in same species or same genus.

| Taxon1 | Taxon2 | Accession1 | Accession2 | % of identity | Taxon1 | Taxon2 | Accession1 | Accession2 | % of identity |
| --- | --- | --- | --- | --- | --- | --- | --- | --- | --- |
| *Equisetum arvense* | *Equisetum arvense* | NC_014699 | JN968380 | 98.86 | *Deparia pycnosora* | *Deparia viridifrons* | NC_035845 | NC_035846 | 96.92 |
| *Mankyua chejuensis* | *Mankyua chejuensis* | NC_017006 | KP205433 | 99.97 | *Diplaziopsis cavaleriana* | *Diplaziopsis javanica* | NC_035847 | NC_035848 | 96.36 |
| *Psilotum nudum* | *Psilotum nudum* | NC_003386 | KC117179 | 99.71 | *Diplazium bellum* | *Diplazium dilatatum* | NC_035849 | NC_035850 | 93.87 |
| *Lygodium japonicum* | *Lygodium japonicum* | NC_022136 | KF225593 | 99.89 | *Diplazium bellum* | *Diplazium dushanense* | NC_035849 | NC_035851 | 93.51 |
| *Azolla caroliniana* | *Azolla caroliniana* | MF177092 | MF177093 | 99.89 | *Diplazium bellum* | *Diplazium striatum* | NC_035849 | NC_035852 | 93.59 |
| *Pteris vittata* | *Pteris vittata* | MH500228.1 | NC_040212.1 | 99.97 | *Diplazium bellum* | *Diplazium unilobum* | NC_035849 | NC_035853 | 93.09 |
| *Deparia lancea* | *Deparia lancea* | NC_035844 | MH124235.1 | 99.74 | *Diplazium dilatatum* | *Diplazium dushanense* | NC_035850 | NC_035851 | 93.16 |
| *Macrothelypteris torresiana* | *Macrothelypteris torresiana* | NC_035858 | MH500230.1 | 99.97 | *Diplazium dilatatum* | *Diplazium striatum* | NC_035850 | NC_035852 | 97.53 |
| *Equisetum arvense* | *Equisetum hyemale* | NC_014699 | NC_020146 | 86.89 | *Diplazium dilatatum* | *Diplazium unilobum* | NC_035850 | NC_035853 | 92.48 |
| *Angiopteris evecta* | *Angiopteris angustifolia* | NC_008829 | NC_026300 | 98.12 | *Diplazium dushanense* | *Diplazium striatum* | NC_035851 | NC_035852 | 92.92 |
| *Hymenophyllum holochilum* | *Hymenophyllum coreanum* | NC_039753.1 |  | 81.02 | *Diplazium dushanense* | *Diplazium unilobum* | NC_035851 | NC_035853 | 93.72 |
| *Hymenophyllum holochilum* | *Hymenophyllum wrightii* | NC_039753.1 |  | 84.95 | *Diplazium striatum* | *Diplazium unilobum* | NC_035852 | NC_035853 | 92.19 |
| *Hymenophyllum holochilum* | *Hymenophyllum polyanthos* | NC_039753.1 |  | 81.2 | *Woodsia macrochlaena* | *Woodsia polystichoides* | NC_035864 | NC_035865 | 96.22 |
| *Hymenophyllum holochilum* | *Hymenophyllum barbatum* | NC_039753.1 |  | 92.02 | *Cyrtomium devexiscapulae* | *Cyrtomium falcatum* | NC_028542 | NC_028705 | 99.88 |
| *Hymenophyllum wrightii* | *Hymenophyllum barbatum* |  |  | 84.62 | *Cyrtomium devexiscapulae* | *Cyrtomium fortunei* | NC_028542 | NC_037510.1 | 99.92 |
| *Hymenophyllum wrightii* | *Hymenophyllum polyanthos* |  |  | 89.18 | *Cyrtomium falcatum* | *Cyrtomium fortunei* | NC_028705 | NC_037510.1 | 99.83 |
| *Hymenophyllum wrightii* | *Hymenophyllum coreanum* |  |  | 90.03 | *Dryopteris blanfordii* | *Dryopteris crassirhizoma* | LT827127.1 | MK554795.1 | 97.78 |
| *Hymenophyllum barbatum* | *Hymenophyllum polyanthos* |  |  | 82.26 | *Dryopteris blanfordii* | *Dryopteris decipiens* | LT827127.1 | NC_035854.1 | 91.13 |
| *Hymenophyllum barbatum* | *Hymenophyllum coreanum* |  |  | 83.01 | *Dryopteris blanfordii* | *Dryopteris filix-mas* | LT827127.1 | LT618774.1 | 95.35 |
| *Hymenophyllum polyanthos* | *Hymenophyllum coreanum* |  |  | 93.35 | *Dryopteris blanfordii* | *Dryopteris villarii* | LT827127.1 | LT905144.1 | 95.05 |
| *Lygodium japonicum* | *Lygodium microphyllum* | NC_022136 | NC_039378.1 | 89.69 | *Dryopteris crassirhizoma* | *Dryopteris decipiens* | MK554795.1 | NC_035854.1 | 91.43 |
| *Alsophila podophylla* | *Alsophila spinulosa* | NC_038150.1 | NC_012818 | 86.16 | *Dryopteris crassirhizoma* | *Dryopteris filix-mas* | MK554795.1 | LT618774.1 | 95.54 |
| *Adiantum aleuticum* | *Adiantum capillus-veneris* | NC_040209.1 | NC_004766 | 80.05 | *Dryopteris crassirhizoma* | *Dryopteris villarii* | MK554795.1 | LT905144.1 | 95.39 |
| *Adiantum aleuticum* | *Adiantum hispidulum* | NC_040209.1 | LT615217.1 | 81.04 | *Dryopteris decipiens* | *Dryopteris filix-mas* | NC_035854.1 | LT618774.1 | 93.51 |
| *Adiantum aleuticum* | *Adiantum shastense* | NC_040209.1 | NC_037478.1 | 91.98 | *Dryopteris decipiens* | *Dryopteris villarii* | NC_035854.1 | LT905144.1 | 93.66 |
| *Adiantum capillus-veneris* | *Adiantum hispidulum* | NC_004766 | LT615217.1 | 80.21 | *Dryopteris filix-mas* | *Dryopteris villarii* | LT618774.1 | LT905144.1 | 97.98 |
| *Adiantum capillus-veneris* | *Adiantum shastense* | NC_004766 | NC_037478.1 | 83.12 | *Azolla caroliniana* | *Azolla filiculoides* | MF177092 | MF177094 | 96.78 |
| *Adiantum hispidulum* | *Adiantum shastense* | LT615217.1 | NC_037478.1 | 84.17 | *Azolla caroliniana* | *Azolla mexicana* | MF177092 | MF177088 | 97.39 |
| *Cheilanthes lindheimeri* | *Cheilanthes micropteris* | NC_014592 | NC_040174.1 | 84.29 | *Azolla caroliniana* | *Azolla microphylla* | MF177092 | MF177089 | 97.37 |
| *Ceratopteris cornuta* | *Ceratopteris richardii* | NC_040204.1 | KM052729 | 98.62 | *Azolla caroliniana* | *Azolla nilotica* | MF177092 | MF177090 | 86.51 |
| *Myriopteris covillei* | *Myriopteris scabra* | NC_039724.1 | NC_040213.1 | 85.02 | *Azolla caroliniana* | *Azolla rubra* | MF177092 | MF177091 | 96.77 |
| *Vittaria appalachiana* | *Vittaria graminifolia* | NC_040219.1 | NC_040217.1 | 96.67 | *Azolla filiculoides* | *Azolla mexicana* | MF177094 | MF177088 | 96.29 |
| *Asplenium pekinense* | *Asplenium prolongatum* | NC_035837 | NC_035838 | 85.39 | *Azolla filiculoides* | *Azolla microphylla* | MF177094 | MF177089 | 96.28 |
| *Athyrium anisopterum* | *Athyrium opacum* | NC_035738 | NC_035841 | 95.58 | *Azolla filiculoides* | *Azolla nilotica* | MF177094 | MF177090 | 86.52 |
| *Athyrium anisopterum* | *Athyrium sheareri* | NC_035738 | NC_035836 | 95.44 | *Azolla filiculoides* | *Azolla rubra* | MF177094 | MF177091 | 99.3 |
| *Athyrium anisopterum* | *Athyrium sinense* | NC_035738 | NC_035839 | 98.24 | *Azolla mexicana* | *Azolla microphylla* | MF177088 | MF177089 | 99.61 |
| *Athyrium opacum* | *Athyrium sheareri* | NC_035841 | NC_035836 | 95.31 | *Azolla mexicana* | *Azolla nilotica* | MF177088 | MF177090 | 86.24 |
| *Athyrium opacum* | *Athyrium sinense* | NC_035841 | NC_035839 | 95.95 | *Azolla mexicana* | *Azolla rubra* | MF177088 | MF177091 | 96.27 |
| *Athyrium sheareri* | *Athyrium sinense* | NC_035836 | NC_035839 | 95.79 | *Azolla microphylla* | *Azolla nilotica* | MF177089 | MF177090 | 86.27 |
| *Cystopteris chinensis* | *Cystopteris protrusa* | NC_035843 | KP136830 | 92.66 | *Azolla microphylla* | *Azolla rubra* | MF177089 | MF177091 | 96.27 |
| *Deparia lancea* | *Deparia pycnosora* | NC_035844 | NC_035845 | 96.81 | *Azolla nilotica* | *Azolla rubra* | MF177090 | MF177091 | 86.51 |
| *Deparia lancea* | *Deparia viridifrons* | NC_035844 | NC_035846 | 95.74 |  |  |  |  |  |


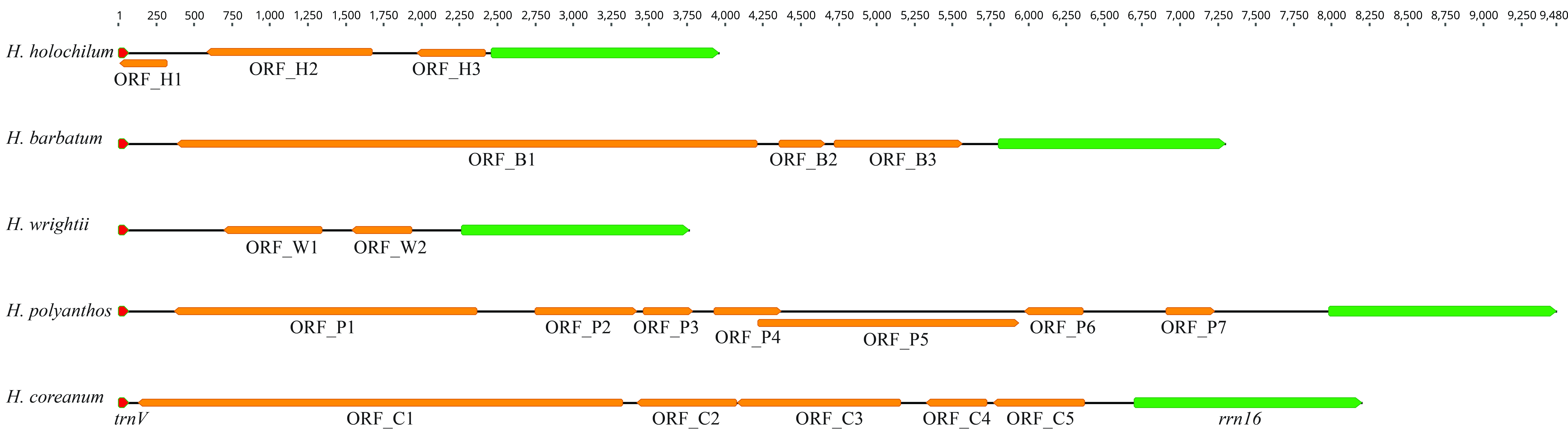


Supplementary figure 1. ORFs between *trnV* (red circle) and *rrn16* (green bar) in five *Hymenphyllum* species.
